# Barriers and Facilitators Associated with Non-Surgical Treatment Use for Osteoarthritis Patients in Orthopaedic Practice

**DOI:** 10.1371/journal.pone.0147406

**Published:** 2016-01-22

**Authors:** Stefanie N. Hofstede, Perla J. Marang-van de Mheen, Thea P. M. Vliet Vlieland, Cornelia H. M. van den Ende, Rob G. H. H. Nelissen, Leti van Bodegom-Vos

**Affiliations:** 1 Department of Medical Decision Making, Leiden University Medical Center, RC Leiden, The Netherlands; 2 Department of Orthopaedics, Leiden University Medical Center, RC Leiden, The Netherlands; 3 Department of Rheumatology, Sint Maartenskliniek Nijmegen, Hengstdal 3, Ubbergen, The Netherlands; Glasgow University, UNITED KINGDOM

## Abstract

**Introduction:**

International evidence-based guidelines for the management of patients with hip and knee osteoarthritis (OA) recommend to start with (a combination of) non-surgical treatments, and using surgical intervention only if a patient does not respond sufficiently to non-surgical treatment options. Despite these recommendations, there are strong indications that non-surgical treatments are not optimally used in orthopaedic practice. To improve the adoption of non-surgical treatments, more insight is needed into barriers and facilitators of these treatments. Therefore, this study assessed which barriers and facilitators are associated with the use and prescription of different non-surgical treatments before hip and knee OA in orthopaedic practice among patients and orthopaedic surgeons in the Netherlands.

**Materials and Methods:**

We performed two internet-based surveys among 172 orthopaedic surgeons and 174 OA patients. Univariate association and multivariable regression techniques are used to identify barriers and facilitators associated with the use of non-surgical treatments.

**Results:**

Most barriers and facilitators among patients were associated with the use of physical therapy, lifestyle advice and dietary therapy. Among orthopaedic surgeons, most were associated with prescription of acetaminophen, dietary therapy and physical therapy. Examples of barriers and facilitators among patients included “People in my environment had positive experiences with a surgery” (facilitator for education about OA), and “Advice of people in my environment to keep on moving” (facilitator for lifestyle and dietary advice). For orthopaedic surgeons, examples were “Lack of knowledge about guideline” (barrier for lifestyle advice), “Agreements/ deliberations with primary care” and “Easy communication with a dietician” (facilitators for dietary therapy). Also the belief in the efficacy of these treatments was associated with increased prescription.

**Conclusions:**

Strategies to improve non-surgical treatment use in orthopaedic practice should be targeted at changing the beliefs of orthopedic surgeons, communication with other OA care providers and involving patient’s environment in OA treatment.

## Introduction

Patients with symptomatic knee or hip osteoarthritis (OA) may suffer from pain and loss of function, which can be treated by performing a total hip arthroplasty (THA) or total knee arthroplasty (TKA). However, these treatments should not be given too early, given the limited lifespan of a prosthesis and the less successful outcomes after revision than after primary THA or TKA [1]. Therefore, international evidence-based guidelines for hip and knee OA recommend to start with (a combination of) non-surgical treatments [2–6]. These treatments aim to prevent progression and reduce symptoms such as joint pain and impairment of functions [6]. Following the existing guidelines in the Netherlands, patients with OA are first treated by the general practitioner and referred to an orthopedic surgeon if they do not respond sufficiently to non-surgical treatment options. In orthopaedic practice, the decision will be made to continue non-surgical treatments or to perform surgery. A stepped-care strategy (SCS) based on (inter)national guidelines [7,8] was developed to facilitate the use of non-surgical treatments in three steps.

Despite recommendations and the SCS, previous studies suggest that the use of non-surgical treatments in patients with hip or knee OA can be improved [9–12]. For example, Snijders et al. [9] found that 81% of patients with hip or knee OA did not receive all non-surgical treatments in the primary care setting. Many patients may thus be referred to orthopaedic practice without having received all recommended non-surgical options. In these cases, the orthopaedic surgeon could provide additional non-surgical treatments. However, our previous study showed that only 10% of the patients in orthopaedic practice received all recommended non-surgical treatments before surgery [13]. These findings are consistent with the rising number of THA and TKA in OA patients in the Netherlands [14]. In addition, the large variation in preoperative status (e.g. disease severity) across different centers in Europe and Australia [15,16] suggests differences in timing of surgery, possibly influenced by non-surgical treatment use. An improved use of non-surgical treatments may reduce surgery rates as well as variation in preoperative status.

More insight is needed into factors that hinder (barriers) and facilitate (facilitators) the use of recommended non-surgical treatments in orthopaedic practice. Some studies have been carried out focused at identifying barriers or facilitators for a specific non-surgical treatment, [17–19], or carried out in primary care [20]. However, it is unknown whether identified factors in these studies also apply to non-surgical treatment use in orthopaedic practice. Furthermore, previous research mainly focused on barriers and facilitators at the patient level [10,21], such as people's own perceptions of the need to seek treatment [22]. However, barriers or facilitators may exist among professionals or within organizations that influence non-surgical treatment use.

Therefore, the aim of the study is to assess which barriers and facilitators are associated with the use and prescription of different non-surgical treatments before hip or knee osteoarthritis (OA) in orthopaedic practice among patients and orthopaedic surgeons.

## Materials and Methods

### Study design

Cross-sectional internet-based surveys among OA patients and orthopaedic surgeons.

### Survey development

To identify potential barriers and facilitators for non-surgical treatment use, semi-structured interviews were performed among 10 orthopaedic surgeons involved in hip and knee surgery and 5 patients in whom TKA or THA was performed no longer than 12 months ago. Purposive sampling was applied to obtain contrasting views and thereby identify all potential barriers and facilitators. Therefore, patients and orthopaedic surgeons were selected from Dutch regions with high and low surgery rates based on the report of Van Beek et al. [23], as participants from regions with lower surgery rates may perceive more facilitators and participants from regions with higher surgery rates more barriers. Furthermore, we selected participants from both academic and non-academic hospitals to take the possible impact of a different organization of care into account.

The interview questions were formulated to ensure the representation of all levels of the framework of Grol and Wensing [24] and the constructs of the Theoretical Domains Interview framework (TDI)[25]. The framework of Grol and Wensing distinguishes the following levels: the innovation, the professional, the patient, the social context, the organizational context, and the external environment (political and economic factors) [24]. The TDI framework includes 12 theoretical construct domains derived from 33 psychological theories and covering 128 explanatory constructs that enhance implementation of evidence-based practice[25]. In addition, previously reported barriers and facilitators in primary care [26] were included. The semi-structured interviews were audio-taped, transcribed in full and analyzed using open coding. The qualitative analysis was executed using the software package ATLAS.ti (ATLAS.ti Scientific Software Development GmBH, Berlin, Germany). A total of 35 barriers and 23 facilitators were identified during the in-depth interviews among orthopaedic surgeons and 20 barriers and 12 facilitators among patients.

### Survey for patients

#### Population

The developed internet-based survey was sent to a sample of patients (n = 195), to estimate a previously reported 19% use of non-surgical treatments among 47,000 patients with hip and knee OA annually in the Netherlands, with a 5% margin of error [13,27]. Patients were recruited via advertisements in newspapers, and at websites or newsletters of patient associations. Inclusion criteria were: age ≥ 18 years, a doctor’s diagnosis of hip or knee OA, and either having TKA or THA performed no longer than 12 months ago or being on the waiting list for surgery within three months. The latter criteria were included to ensure that the decision for surgery had already been made. Patients with an inability to understand written Dutch or who had undergone revision surgery were excluded. Two reminders were sent in case of non-response, one after 1.5 weeks and again after three weeks. Participants received a ten euro gift card as an incentive upon completion of the questionnaire.

#### Survey

The first part of the survey included questions on patient characteristics: age, gender, region of residence (north, middle, and south), educational level (basic education (no or only primary education), intermediate education (prevocational secondary education, senior secondary vocational training, senior secondary general education, pre-university education), or higher education (higher professional education or university (bachelor, master, or PhD degree)), work situation (paid work or no paid work), height and weight to calculate the Body Mass Index (BMI), and type of insurance (basic coverage and additional coverage). All inhabitants of the Netherlands have a basic insurance coverage (legally obliged) and have the option of purchasing supplementary insurance covering additional healthcare such as physical therapy and dietary therapy, rather than being subject to out of pocket expenses. In addition, questions were included about use of each of the recommended non-surgical options (education about OA, education about different treatment options, lifestyle advice, dietary therapy, physical therapy, acetaminophen, NSAIDs, and glucocorticoid injections (only for knee OA) [28]) which were formulated as follows: “Did you receive the following treatments for your complaints on your affected joint before the joint replacement surgery?” (yes/ no). The second part of the questionnaire consisted of 32 items covering the identified barriers and facilitators from the interviews. Patients were asked to indicate the influence of each facilitator and barrier on non-surgical treatment use. Answers could be given on a 4-point Likert scale ranging from not important to very important, or to indicate “not applicable to my situation” for example for the facilitator “guidance of the exercise therapist” if the patient had never visited one. The survey was pilot tested among three patients to test whether patients understood the questions and answering categories.

### Survey for orthopaedic surgeons

#### Population

All 482 Dutch orthopaedic surgeons listed with an email address in the registry of the Dutch Orthopaedic Association (NOV) and/or the Dutch medical address book were invited to participate. Inclusion criterion was: seeing patients with hip or knee OA. Non-responders received two reminders, one after 1.5 weeks and again after three weeks.

#### Survey

The first part of the survey included questions about background characteristics: age, gender, work region, work setting, years of working experience, number of new patients with hip/ knee OA per month. In addition, questions were included about prescription of each of the recommended non-surgical options and were formulated as follows: “If patients did not receive the following non-surgical treatments, do you prescribe these treatments?” In case of physical therapy and dietary therapy we asked whether they referred patients, rather than prescribe these treatments themselves. Answers could be given on a 4-point scale ranging from never to almost always. The second part of the questionnaire consisted of 58 items covering the identified barriers and facilitators from the interviews. Orthopaedic surgeons were asked to what degree each barrier and facilitator influenced the prescription of non-surgical treatments in patients with hip and/ or knee OA. Answers could be given on a 4-point Likert scale ranging from none to a large extent.

### Analysis

Data from all respondents completing the survey and fulfilling the inclusion criteria were included in the analyses. Descriptive statistics showed that many patients reported barriers and facilitators as not applicable to their situation, even though a number of these seem to be applicable to any patients' situation, *e*.*g*., "The practitioner took my problem seriously". As each patient visited a practitioner, all patients should have been able to answer this question but this was not the case. Given this example, we assume that patients have misunderstood “not applicable” as “not important”, and that they selected an item as important only if they felt strongly about it. It was included accordingly in the analyses. We performed a sensitivity analysis treating the answers “not applicable” as missing in the univariate analyses. In addition, barriers and facilitators for patients were dichotomized into not important (grouping answering categories not important/ a little bit important/ not applicable on my situation) and important (grouping the answering categories important/ very important), because of few observations in some cells.

For patients, we first assessed the extent to which each barrier/ facilitator was associated with the use of each non-surgical treatment using univariate logistic regression analysis, with the barrier/facilitator (not important/ important) as the independent variable and use of each recommended treatment (yes/ no) as dependent variable. For orthopaedic surgeons this was done using the Spearman rank correlation as both the independent variable (influence of barrier/ facilitator for prescription of non-surgical treatments) and the dependent variable (prescription of the different non-surgical treatments) consisted of Likert scales with a clear ordering, but without information on the distance between the 4 points on the scale.

Secondly, as individual barriers/ facilitators may be related to others we included barriers/ facilitators significantly associated with use of each non-surgical treatment into a multivariable logistic regression model (p<0.05). Given the multiple testing in the first step, we used the more conservative p-value of 0.05 to include barriers/ facilitators in the multivariable model, rather than the commonly used o-value of 0.10 or 0.20. For orthopaedic surgeons, we dichotomized prescribed non-surgical treatments into “provided” (often/ almost always) and “not provided” (never/ sometimes) and barriers and facilitators into “0” (not at all/ a little bit) and “1” (to a reasonable extent/ to a large extent), because of few observations in some cells. All analyses were executed using the software package SPSS (IBM SPSS Statistics, version 20.0).

### Ethics Statement

The Medical Ethical Committee (CME P13.087/NV/nv) confirmed that ethical approval for this type of study is not required under Dutch law.

## Results

### Response and characteristics of the population

Of the 195 recruited patients, 8 did not fulfill the inclusion criteria because they did not receive a surgery in the last 12 months and were not on the waiting list to receive surgery within 3 months. Of the remaining 187 patients, 174 (93%) completed the questionnaire. Nine of the 482 orthopaedic surgeons were excluded because they did not see patients with OA in consultations and 172 (36%) completed the questionnaire. Patients who responded had an average age of 64 (SD 7.7), were mostly female (72%), overweight (78%), and intermediate educational level (69%). Five patients (3%) were still on the waiting list, the remaining 169 patients had received a joint replacement. For 73% of the 174 patients it was their first joint replacement, 54% received a total knee and the history of complaints was less than 1 year for 8%, 1–5 years for 49% and more than 5 years for 43% of the patients. Patients reported a median pain score of 8.0 before surgery on a 0 (no pain)-10 (unbearable pain) Likert scale. Almost all patients had additional insurance coverage, meaning that physical and dietary therapy was also (partly) covered by their insurance rather than being subject to out of pocket expenses.

Orthopaedic surgeons had an average age of 48.4 (SD 8.6), were mostly males (91%), had worked on average 12.8 (SD 8.0) years as an orthopaedic surgeon, and saw on average 25.1 (SD 22.2) new patients with hip OA and 31.3 (SD 23.9) patients with knee OA per month. The majority worked at a general hospital (52%). Both patients and orthopaedic surgeons were spread across different regions of the Netherlands.

### Barriers and facilitators among patients for non-surgical treatment use

Table 1 shows all barriers (-) and facilitators (+) in the survey for each level of the framework of Grol and Wensing [24] and whether patients considered these barriers and facilitators as important. Most patients reported the following facilitators as very important: “Important to exercise/ to keep on moving at home” (50.6%), “Guidance by the physical therapist” (36.8%) and “Sufficient time of the practitioner(s) to explain everything” (31.6%). Barriers reported by most patients as very important were: “Preference of practitioner for surgery” (31.6%), “Too much loss of cartilage to use non-surgical treatments” (29.9%) and “People in my environment had positive experiences with a surgery” (26.4%).

**Table 1:** The importance of barriers and facilitators reported by patients for non-surgical treatment use.

Barriers and facilitators	Not important n (%)	A little bit important n (%)	Important n (%)	Very important n (%)	Not applicable n (%)
**Innovation**					
**Individual professional**					
Guidance by the physical therapist (+)	5 (2.9)	8 (4.6)	53 (30.5)	64 (36.8)	44 (25.3)
The practitioner(s) took my problem serious (+)	8 (4.6)	10 (5.7)	64 (36.8)	38 (21.8)	54 (31)
Sufficient information about non-surgical treatments (+)	2 (1.1)	12 (6.9)	63 (36.2)	36 (20.7)	61 (35.1)
Preference of practitioner(s) for non-surgical treatments (+)	1 (0.6)	10 (5.7)	66 (37.9)	33 (19.0)	64 (36.8)
The orthopaedic surgeon asked about previously received treatments (+)	10 (5.7)	7 (4.0)	40 (23.0)	29 (16.7)	88 (50.6)
Because of the good contact with my treating practitioner(s), I was able to carry on with non-surgical treatments (+)	10 (5.7)	4 (2.3)	75 (43.1)	25 (14.4)	60 (34.5)
Explanation of drawbacks of the surgery (+)	8 (4.6)	11 (6.3)	42 (24.1)	24 (13.8)	89 (51.1)
Preference of practitioner for surgery (-)	11 (6.3)	5 (2.9)	38 (21.8)	55 (31.6)	65 (37.4)
Mainly the benefits of a surgery were discussed (-)	11 (6.3)	14 (8.0)	29 (16.7)	34 (19.5)	86 (49.4)
Lack of information provision about the use of acetaminophen (-)	21 (12.1)	6 (3.4)	15 (8.6)	19 (10.9)	113 (64.9)
Lack of information provision about the use of injections (-)	19 (10.9)	2 (1.1)	15 (8.6)	19 (10.9)	119 (68.4)
Lack of information provision about physical therapy (-)	16 (9.2)	6 (3.4)	14 (8.0)	18 (10.3)	120 (69.0)
The practitioner did not think physical therapy was necessary (-)	12 (6.9)	4 (2.3)	14 (8.0)	18 (10.3)	126 (72.4)
Lack of information provision about the use of NSAIDs (-)	16 (9.2)	8 (4.6)	11 (6.3)	13 (7.5)	126 (72.4)
Lack of information provision by my practitioner (-)	15 (8.6)	11 (6.3)	20 (11.5)	10 (5.7)	118 (67.8)
Lack of empathy of the practitioner (-)	20 (11.5)	8 (4.6)	13 (7.5)	8 (4.6)	125 (71.8)
Lack of guidance with weight loss (-)	19 (10.9)	2 (1.1)	9 (5.2)	7 (4.0)	137 (78.7)
**Patient**					
Important to exercise/ to keep on moving at home (+)	1 (0.6)	6 (3.4)	58 (33.3)	88 (50.6)	21 (12.1)
Surgery was the last treatment option (+)	8 (4.6)	10 (5.7)	42 (24.1)	49 (28.2)	65 (37.4)
Too much loss of cartilage to use non-surgical treatments (-)	13 (7.5)	11 (6.3)	39 (22.4)	52 (29.9)	59 (33.9)
Too much pain (-)	16 (9.2)	12 (6.9)	39 (22.4)	43 (24.7)	64 (36.8)
I prefer not to use medication (-)	15 (8.6)	9 (5.2)	38 (21.8)	38 (21.8)	74 (42.5)
I cannot do anything to prevent/slow the development of OA (-)	21 (12.1)	11 (6.3)	26 (14.9)	33 (19.0)	83 (47.7)
Comorbidities (-)	14 (8.0)	4 (2.3)	9 (5.2)	14 (8.0)	134 (76.4)
Dissatisfaction with physical therapy (-)	22 (12.6)	3 (1.7)	9 (5.2)	10 (5.7)	130 (74.7)
Negative attitude towards exercises (-)	28 (16.1)	5 (2.9)	16 (9.2)	10 (5.7)	115 (66.1)
Lack of trust in non-surgical treatments (-)	19 (10.9)	8 (4.6)	16 (9.2)	9 (5.2)	122 (70.1)
**Social context**					
Advice of people in my environment to keep on moving (+)	12 (6.9)	12 (6.9)	52 (29.9)	38 (21.8)	60 (34.5)
Good collaboration between the practitioners (+)	6 (3.4)	14 (8.0)	57 (32.8)	20 (11.5)	77 (44.3)
People in my environment had positive experiences with surgery (-)	10 (5.7)	9 (5.2)	46 (26.4)	46 (26.4)	63 (36.2)
**Organizational context**					
Sufficient time of the practitioner(s) to explain everything (+)	2 (1.1)	8 (4.6)	76 (43.7)	55 (31.6)	33 (19.0)
**Economic and political context**					
Additional payment for physical therapy not (fully) covered by insurance (-)	18 (10.3)	3 (1.7)	5 (2.9)	14 (8.0)	134 (77.0)

(+) Mentioned as facilitator in the interviews, asked as facilitator in the questionnaire

(-) Mentioned as barrier in the interviews, asked as barrier in the questionnaire

Table 2 shows univariate associations between barriers/ facilitators and non-surgical treatments. Physical therapy, lifestyle advice and dietary therapy were associated with the largest numbers of barriers and facilitators *e*.*g*. a higher use of physical therapy and dietary therapy was associated with “Because of the good contact with my treating practitioner(s), I was able to carry on with non-surgical treatments” OR 5.68 (95% CI 2.71–11.93) and OR 4.17 (95% CI 1.33–13.07), respectively. A higher “use” of lifestyle advice was associated with “Important to exercise/ to keep on moving at home” (OR 6.52 (95% CI 2.59–16.43)). Treating the answers “not applicable” as missing gave similar results in the univariate analyses (data not shown).

**Table 2:** The influence of barriers and facilitators reported by patients for non-surgical treatment use (univariate analyses).

	Education about OA OR (95% CI)	Education about different treatment options OR (95% CI)	Lifestyle advice OR (95% CI)	(Referral to) dietary therapy OR (95% CI)	Physical therapy OR (95% CI)	Acetaminophen OR (95% CI)	NSAIDs OR (95% CI)
**Innovation**							
**-**							
**Individual professional**							
Guidance by the physical therapist (+)	**2.34 (1.10–4.99)**	**2.39 (1.24–4.63)**	**3.76 (1.94–7.32)**	1.90 (0.65–5.54)	**24.00 (10.00-57-58)**	1.85 (0.93–3.68)	1.30 (0.68–2.50)
The practitioner(s) took my problem serious (+)	1.93 (0.91–4.07)	**2.22 (1.17–4.41)**	**3.47 (1.83–6.59)**	2.61 (0.90–7.56)	**3.57 (1.77–7.20)**	1.54 (0.79–3.00)	1.654 (0.88–3.10)
Sufficient information about non-surgical treatments (+)	**2.75 (1.28–5.91)**	**3.05 (1.59–5.84)**	**3.39 (1.79–6.41)**	**3.04 (1.05–8.79)**	**7.39 (3.41–16.00)**	1.24 (0.64–2.41)	**2.00 (1.07–3.75)**
Preference of practitioner(s) for non-surgical treatments (+)	**2.36 (1.11–5.04)**	**3.41 (1.77–6.56)**	**3.39 (1.79–6.41)**	**2.93 (1.01–1.42)**	**8.67 (3.91–19.19)**	1.24 (0.64–2.41)	**2.22 (1.18–4.17)**
The orthopaedic surgeon asked about previously received treatments (+)	**2.64 (1.12–6.22)**	**2.30 (1.16–4.54)**	**1.99 (1.04–3.79)**	**3.02 (1.18–7.75)**	**4.51 (1.95–10.40)**	**2.99 (1.40–6.38)**	1.90 (0.97–3.66)
Because of the good contact with my treating practitioner(s), I was able to carry on with non-surgical treatments (+)	1.81 (0.86–3.83)	1.85 (0.98–3.50)	**3.18 (1.69–6.01)**	**4.17 (1.33–13.07)**	**5.68 (2.71–11.93)**	1.62 (0.83–3.14)	1.384 (0.74–2.58)
Explanation of drawbacks of the surgery (+)	**2.42 (1.03–5.70**	1.63 (0.84–3.18)	**3.58 (1.78–7.21)**	**4.51 (1.70–11.95)**	**4.10 (1.77–9.46)**	**3.17 (1.45–6.90)**	1.70 (0.88–3.28)
Lack of information provision about the use of acetaminophen (-)	1.59 (0.56–4.44)	1.09 (0.49–2.43)	0.65 (0.30–1.37)	1.45 (0.51–4.11)	**0.44 (0.20–0.97)**	1.35 (0.56–3.22)	1.46 (0.65–3.30)
The practitioner did not think physical therapy was necessary (-)	2.81 (0.40–9.84)	0.83 (0.37–1.83)	0.90 (0.41–1.96)	**4.65 (1.68–12.87)**	0.55 (0.24–1.23)	1.50 (0.60–3.74)	1.31 (0.58–2.98)
Lack of information provision by my practitioner (-)	2.57 (0.73–9.03)	1.51 (0.63–3.63)	0.93 (0.42–2.07)	2.91 (0.95–7.61)	1.26 (0.50–3.18)	1.35 (0.54–3.39)	**2.62 (1.01–6.81)**
Lack of empathy of the practitioner (-)	5.71 (0.74–44.13)	1.03 (0.39–2.71)	1.29 (0.49–3.38)	1.46 (0.37–5.77)	**8.60 (1.12–65.99)**	4.21 (0.94–18.82)	1.48 (0.55–4.04)
Lack of guidance with weight loss (-)	4.11 (0.52–32.25)	1.60 (0.49–5.20)	2.95 (0.81–10.77)	**5.41 (1.73–16.99)**	6.16 (0.79–48.01)	6.55 (0.84–50.97)	1.79 (0.55–5.80)
**Patient**							
Important to exercise/ to keep on moving at home (+)	1.10 (0.41–4.96)	1.88 (0.83–4.28)	**6.52 (2.59–16.43)**	5.06 (0.64–5.06)	**5.98 (2.53–14.10)**	1.84 (0.79–4.28)	1.66 (0.74–3.77)
Surgery was the last treatment option (+)	**3.49 (1.56–7.83)**	**2.82 (1.47–5.40)**	**3.71 (1.95–7.08)**	**3.23 (1.18–8.83)**	**5.57 (2.59–11.98)**	1.51 (0.78–2.94)	**2.23 (1.19–4.19)**
I cannot do anything to prevent/ slow the development of OA (-)	1.96 (0.83–4.63)	1.26 (0.64–2.47)	1.69 (0.87–3.29)	1.97 (0.79–4.91)	0.68 (0.34–1.35)	**2.15 (1.01–4.61)**	1.67 (0.60–2.25)
Negative attitude towards exercises (-)	1.07 (0.37–30.7)	0.79 (0.34–1.87)	1.22 (0.51–2.91)	**2.91 (1.02–8.31)**	0.68 (0.34–1.35)	1.37 (0.51–3.63)	1.65 (0.60–2.25)
Lack of trust in non-surgical treatments (-)	0.77 (0.28–2.09)	**0.34 (0.14–0.81)**	0.93 (0.37–2.1)	1.29 (0.39–4.23)	1.20 (0.45–3.22)	3.28 (0.93–11.50)	1.55 (0.61–3.94)
**Social context**							
Advice of people in my environment to keep on moving (+)	1.56 (0.74–3.30)	1.30 (0.69–2.43)	**4.45 (2.30–8.58)**	**12.91 (2.88–57.82)**	1.47 (0.75–2.89)	1.64 (0.84–3.20)	**1.94 (1.04–3.63)**
Good collaboration between the practitioners (+)	1.97 (0.90–4.34)	1.54 (0.81–2.93)	**2.99 (1.56–5.75)**	**5.80 (2.00–16.81)**	**5.80 (2.51–13.39)**	1.74 (0.88–3.45)	**2.04 (1.07–3.87)**
People in my environment had positive experiences with surgery (-)	**3.60 (1.60–8.06)**	1.71 (0.91–3.22)	1.15 (0.62–2.12)	1.91 (0.75–4.88)	0.61 (0.31–1.21)	1.56 (0.80–3.04)	1.03 (0.56–1.92)
**Organizational context**							
Sufficient time of the practitioner(s) to explain everything (+)	1.29 (0.56–2.95)	1.80 (0.89–3.65)	**4.40 (2.12–9.14)**	**8.97 (1.16–69.50)**	**3.86 (1.85–8.05)**	1.32 (0.63–2.79)	1.78 (0.88–3.59)
**Economic and political context**							
-							

In bold: P-values ≤ 0.05

Only barriers and facilitators with a significant association with at least one non-surgical treatment are reported

Only a few of these barriers and facilitators were independently and significantly associated with non-surgical treatment use in the multivariable logistic regression analysis (Table 3). People in patients’ environment with positive experiences with surgery was associated with an increased use of OA education, lack of trust in non-surgical treatments was associated with a decreased use of education on different treatment options, and advice of people in patients’ environment to keep on moving was associated with increased use of lifestyle advice. For dietary therapy, advice of people in my environment to keep on moving and good collaboration between the practitioners were associated with an increased use. Guidance by the physical therapist increased the use of physical therapy where lack of information provision about the use of acetaminophen was associated with a decreased use.

**Table 3:** The independent effect of barriers and facilitators reported by patients for non-surgical treatment use (multivariable analyses).

Non-surgical treatment	Used, yes (%)	Barrier (B) or facilitator (F)	Odds ratio (95% Confidence interval)	p-value
Education about OA	80	People in my environment had positive experiences with surgery (-)	3.42 (1.48–7.09)	0.004
Education about different treatment options	66	Lack of trust in non-surgical treatments (-)	0.28 (0.11–0.71)	0.008
Lifestyle advice	61	Advice of people in my environment to keep on moving (+)	3.11 (1.43–6.74)	0.004
(Referral to) dietary therapy (when indicated, n = 130)	18	Advice of people in my environment to keep on moving (+)	11.56 (1.90–70.22)	0.008
		Good collaboration between the practitioners (+)	12.12 (1.22–120.73)	0.033
(Referral to) physical therapy	73	Guidance by the physical therapist (+)	20.52 (5.56–75.79)	<0.001
		Lack of information provision about the use of acetaminophen (-)	0.22 (0.06–0.75)	0.016
Acetaminophen	72	-	-	-
NSAIDs	64	-	-	-

Only barriers and facilitators with P-values ≤ 0.05 are shown in the table

### Barriers and facilitators among orthopaedic surgeons for prescription of non-surgical treatments

Table 4 shows all barriers (-) and facilitators (+) in the survey for each level of the framework of Grol and Wensing [4] and whether orthopaedic surgeons considered these barriers and facilitators as important for the prescription of non-surgical treatments. Facilitators that influenced the prescription of non-surgical treatment to a large extent according to orthopaedic surgeons were: “Important to follow guidelines” (49.4%), “Important to try non-surgical treatments first” (49.4%) and “Acetaminophen has only a few side effects” (48.8%) (Table 4). Barriers reported by most orthopedic surgeons were “Glucocorticoid injections is a symptomatic treatment” (14.0%), “No effect of physical therapy when there is an obvious loss of cartilage” (9.9%) and “Physical therapy for hip OA is not effective” (6.4%).

**Table 4:** The degree of influence of barriers and facilitators reported by orthopaedic surgeons for prescription of non-surgical treatments.

Barriers and facilitators	Not at all n (%)	A little bit n (%)	To a reasonable extent n (%)	To a large extent n (%)
**Innovation**				
Clear referral criteria/ guideline (+)	7 (4.1)	10 (5.8)	96 (55.8)	59 (34.3)
The guideline is outdated (-)	69 (40.1)	74 (43.0)	21 (12.2)	8 (4.7)
Lack of guidance in guideline (-)	54 (31.4)	83 (48.3)	32 (18.6)	3 (1.7)
The guideline is unclear about NSAID dosage (-)	73 (42.4)	64 (37.2)	32 (18.6)	3 (1.7)
**Individual professional**				
Important to follow guidelines (+)	5 (2.9)	3 (1.7)	79 (45.9)	85 (49.4)
Important to try non-surgical treatments first (+)	5 (2.9)	10 (5.8)	72 (41.9)	85 (49.4)
Acetaminophen has only a few side effects (+)	9 (5.2)	20 (11.6)	59 (34.3)	84 (48.8)
Only few drawbacks for the use of non-surgical treatments (+)	2 (1.2)	10 (5.8)	83 (48.3)	77 (44.8)
Patients benefit from weight loss (+)	3 (1.7)	25 (14.5)	82 (47.7)	62 (36.0)
Non-surgical treatments motivate patients to do things themselves (+)	3 (1.7)	44 (25.6)	86 (50.0)	39 (22.7)
Good results of physical therapy (+)	6 (3.5)	57 (33.1)	84 (48.8)	25 (14.5)
Patients benefit from Acetaminophen (+)	6 (3.5)	55 (32.0)	87 (50.6)	24 (14.0)
Important to delay a surgery as long as possible (+)	6 (3.5)	55 (32.0)	87 (50.6)	24 (14.0)
Patients benefit from Glucocorticoid injections (+)^a^	7 (4.1)	62 (36.0)	84 (48.8)	19 (11.0)
Patients benefit from NSAIDs (+)	3 (1.7)	43 (25.0)	109 (63.4)	17 (9.9)
Surgery has many disadvantages/ complications/ risks (+)	17 (9.9)	97 (56.4)	48 (27.9)	10 (5.8)
Total knee arthroplasty leads to little results (+)	91 (52.9)	63 (36.6)	16 (9.3)	2 (1.2)
Glucocorticoid injections is a symptomatic treatment (-)^a^	64 (37.2)	53 (30.8)	31 (18.0)	24 (14.0)
No effect of physical therapy when there is an obvious loss of cartilage (-)	50 (29.1)	59 (34.3)	46 (26.7)	17 (9.9)
Physical therapy for hip OA is not effective (-)	39 (22.7)	74 (43.0)	48 (27.9)	11 (6.4)
Limited results of dietary advice/ weight loss (-)	30 (17.4)	91 (52.9)	40 (23.3)	11 (6.4)
Lack of knowledge about guideline (-)	83 (48.3)	63 (36.6)	21 (12.2)	5 (2.9)
Disagreement with (part of) the guideline (-)	73 (42.4)	79 (45.9)	16 (9.3)	4 (2.3)
Side effects/ contraindications NDAIDs (-)	26 (15.1)	91 (52.9)	53 (30.8)	2 (1.2)
Side effects/ complications of Glucocorticoid injections (-)	57 (33.1)	89 (51.7)	25 (14.5)	1 (0.6)
Reduced success rate of TKA/ THA when surgery is delayed (-)	115 (66.9)	41 (23.8)	15 (8.7)	1 (0.6)
Preference for surgery (-)	161 (93.6)	9 (5.2)	1 (0.6)	1 (0.6)
**Patient**				
Patient cannot afford absenteeism at work (-)	35 (20.3)	82 (47.7)	44 (25.6)	11 (6.4)
Negative attitude of patients towards lifestyle adjustments (-)	61 (35.5)	74 (43.0)	31 (18.0)	6 (3.5)
Losing weight is a sensitive topic (-)	74 (43.0)	61 (35.5)	31 (18.0)	6 (3.5)
Patients do not want to take pills (-)	51 (29.7)	83 (48.3)	33 (19.2)	5 (2.9)
Patient does not want physical therapy (-)	55 (32.0)	87 (50.6)	26 (15.1)	4 (2.3)
Pressure by patient for surgery (-)	35 (20.3)	94 (54.7)	40 (23.3)	3 (1.7)
The decision to perform surgery is more easily made in elderly patients (-)	63 (36.6)	66 (38.4)	41 (23.8)	2 (1.2)
**Social context**				
Agreements with colleagues about the content of the care trajectory (+)	7 (4.1)	43 (25.0)	90 (52.3)	32 (18.6)
Peer review / audit of professional association (+)	17 (9.9)	38 (22.1)	85 (49.4)	32 (18.6)
Positive attitudes of colleagues about non-surgical treatments (+)	13 (7.6)	59 (34.3)	89 (51.7)	11 (6.4)
Trained to be reluctant with Glucocorticoid injections (-)^a^	91 (52.9)	58 (33.7)	15 (8.7)	8 (4.7)
Social pressure of environment patient (-)	74 (43.0)	71 (41.3)	26 (15.1)	1 (0.6)
Lack of feedback between different disciplines (-)	75 (43.6)	65 (37.8)	27 (15.7)	5 (2.9)
**Organizational context**				
Clarity on what the patient has done at the physical therapist (+)	16 (9.3)	31 (18.8)	95 (55.2)	30 (17.4)
Agreements/ deliberations with primary care (GP, physical therapist, dietician) (+)	18 (10.5)	58 (33.7)	75 (43.6)	21 (12.2)
Presence of an obesity clinic (+)	81 (47.1)	66 (38.4)	22 (12.8)	3 (1.7)
A multidisciplinary meeting (+)	90 (52.3)	63 (36.6)	16 (9.3)	3 (1.7)
Easy communication with a dietician (+)	110 (64)	49 (28.5)	10 (5.8)	3 (1.7)
Lack of visibility into physical therapies (-)	64 (37.2)	75 (43.6)	23 (13.4)	10 (5.8)
Non-surgical treatments take a lot of time (-)	78 (45.3)	69 (40.1)	18 (10.5)	7 (4.1)
Lack of referral structure to dietician (-)	104 (60.5)	49 (28.5)	14 (8.1)	5 (2.9)
Non-surgical treatments belong to primary care (-)	105 (61.0)	46 (26.7)	17 (9.9)	4 (2.3)
Quick patients flow with surgery (-)	131 (76.2)	28 (16.3)	11 (6.4)	2 (1.2)
Pressure for production (-)	148 (86.0)	20 (11.6)	2 (1.2)	2 (1.2)
Lack of referral structure to physical therapist (-)	131 (76.2)	32 (18.6)	8 (4.7)	1 (0.6)
Indication for surgery depends on the length of the waiting list (-)	161 (93.6)	9 (5.2)	1 (0.6)	1 (0.6)
**Economic and political context**				
Availability of non-surgical treatments (+)	6 (3.5)	23 (13.4)	100 (58.1)	43 (25.0)
Physical therapy is not (fully) covered by insurance (-)	69 (40.1)	54 (31.4)	40 (23.3)	9 (5.2)
A consult at a dietician is not covered by insurance (-)	107 (62.2)	41 (23.8)	21 (12.2)	3 (1.7)
Availability of surgeries in other hospitals in the area (-)	128 (74.4)	37 (21.5)	4 (2.3)	3 (1.7)
Financial interest in surgery (-)	157 (91.3)	11 (6.4)	2 (1.2)	2 (1.2)

(+) Mentioned as facilitator in the interviews, asked as facilitator in the questionnaire

(-) Mentioned as barriers in the interviews, asked as barrier in the questionnaire

^a^ Only for patients with knee OA

Table 5 shows that the prescription of acetaminophen, dietary therapy and physical therapy were associated with the largest numbers of barriers and facilitators e.g. a higher use of acetaminophen was associated with the belief that acetaminophen has only a few side effects (*r* = 0.48, P<0.01). A higher prescription of dietary therapy was associated with the presence of an obesity clinic (*r* = 0.36, P<0.01). Lower prescription of physical therapy is associated with the belief that physical therapy for hip OA was not effective (*r* = -0.29, P<0.01).

**Table 5:** Influence of barriers and facilitators reported by orthopaedic surgeons for prescription of non-surgical treatments.

	Education about OA	Education about different treatment options	Lifestyle advice	(Referral to) dietary therapy	Physical therapy	Acetaminophen	NSAIDs	Glucocorticoid injection^a^
**Innovation**								
Clear referral criteria/ guideline	*r* = 0.10, P = 0.20	*r* = 0.12, P = 0.12	*r* = 0.09, P = 0.24	***r* = 0.25, P<0.01**	***r* = 0.15, P = 0.05**	***r* = 0.29, P<0.01**	*r* = 0.10, P = 0.18	*r* = 0.05, P = 0.50
The guideline is outdated	*r* = -0.05, P = 0.51	*r* = -0.06, P = 0.46	*r* = 0.02, P = 0.83	*r* = 0.06, P = 0.48	*r* = 0.05, P = 0.51	*r* = -0.06, P = 0.43	***r* = 0.16, P = 0.04**	*r* = 0.10, P = 0.20
Lack of guidance in guideline	*r* = -0.03, P = 0.69	*r* = -0.06, P = 0.46	*r* = -0.02, P = 0.85	*r* = -0.01, P = 0.82	*r* = -0.04, P = 0.62	*r* = -0.07, P = 0.39	*r* = 0.13, P = 0.09	***r* = 0.16, P = 0.03**
**Individual professional**								
Important to try non-surgical treatments first	***r* = 0.21, P<0.01**	*r* = 0.14, P = 0.08	*r* = 0.12, P = 0.13	***r* = 0.17, P = 0.02**	*r* = 0.08, P = 0.28	***r* = 0.25, P<0.01**	*r* = 0.12, P = 0.13	*r* = 0.05, P = 0.56
Acetaminophen has only a few side effects	*r* = 0.14, P = 0.06	***r* = 0.19, P = 0.01**	***r* = 0.28, P<0.01**	***r* = 0.18, P = 0.02**	*r* = 0.08, P = 0.31	***r* = 0.48, P<0.01**	*r* = 0.09, P = 0.24	*r* = 0.10, P = 0.21
Only few drawbacks for the use of non-surgical treatments	*r* = 0.09, P = 0.24	*r* = 0.10, P = 0.21	***r* = 0.21, P<0.01**	***r* = 0.21, P<0.01**	*r* = 0.12, P = 0.12	***r* = 0.23, P<0.01**	*r* = -0.03, P = 0.66	*r* = 0.06, P = 0.47
Patients benefit from weight loss	*r* = 0.03, P = 0.69	*r* = 0.10, P = 0.19	***r* = 0.21, P<0.01**	***r* = 0.43, P<0.01**	*r* = 0.03, P = 0.66	*r* = 0.11, P = 0.16	*r* = 0.06, P = 0.44	*r*<-0.01, P = 0.99
Non-surgical treatments motivate patients to do things themselves	*r* = 0.14, P = 0.08	*r* = 0.13, P = 0.08	***r* = 0.23, P<0.01**	*r* = 0.13, P = 0.09	*r* = 0.12, P = 0.11	***r* = 0.17, P = 0.02**	*r* = -0.01, P = 0.86	*r* = -0.09, P = 0.26
Good results of physical therapy	*r* = -0.02, P = 0.84	*r* = -0.02, P = 0.76	*r* = 0.12, P = 0.11	***r* = 0.16, P = 0.03**	*r* = 0.53, P<0.01	***r* = 0.22, P<0.01**	*r* = 0.11, P = 0.17	*r*<-0.01, P = 0.95
Patients benefit from Acetaminophen	*r* = 0.02, P = 0.79	*r* = -0.05, P = 0.53	*r* = 0.03, P = 0.66	***r* = 0.22, P<0.01**	*r* = 0.10, P = 0.19	***r* = 0.50, P<0.01**	***r* = 0.20, P<0.01**	*r* = 0.07, P = 0.36
Important to delay a surgery as long as possible	***r* = 0.18, P = 0.02**	***r* = 0.18, P = 0.02**	*r* = 0.09, P = 0.27	*r* = 0.04, P = 0.62	*r* = -0.03, P = 0.75	*r* = 0.15, P = 0.05	*r* = -0.03, P = 0.73	*r* = 0.05, P = 0.50
Patients benefit from Glucocorticoid injections^a^	*r* = -0.02, P = 0.80	*r* = 0.03, P = 0.69	*r* = -0.07, P = 0.39	*r*<0.01, P = 0.97	*r* = -0.01, P = 0.92	***r* = 0.16, P = 0.04**	*r* = 0.14, P = 0.06	***r* = 0.49, P<0.01**
Patients benefit from NSAIDs	*r* = 0.08, P = 0.33	*r* = 0.07, P = 0.34	*r* = 0.08, P = 0.29	*r* = 0.15, P = 0.06	*r* = 0.05, P = 0.53	***r* = 0.20, P = 0.01**	***r* = 0.37, P<0.01**	*r* = 0.03, P = 0.72
Surgery has many disadvantages/ complications/ risks	*r* = 0.06, P = 0.44	*r* = 0.01, P = 0.93	*r* = 0.05, P = 0.55	*r* = 0.06, P = 0.46	*r* = 0.06, P = 0.42	***r* = 0.21, P<0.01**	*r* = -0.02, P = 0.85	*r* = -0.01, P = 0.90
Glucocorticoid injections is a symptomatic treatment^a^	*r* = -0.04, P = 0.61	*r* = -0.10, P = 0.20	*r* = 0.09, P = 0.26	*r* = 0.12, P = 0.11	*r* = 0.08, P = 0.30	*r* = -0.05, P = 0.53	*r* = 0.01, P = 0.87	***r* = -0.25, P<0.01**
No effect of physical therapy when there is an obvious loss of cartilage	*r* = 0.02, P = 0.85	*r* = 0.02, P = 0.79	***r* = -0.19, P = 0.02**	*r* = -0.09, P = 0.23	***r* = -0.30, P<0.01**	***r* = -0.21, P<0.01**	*r* = -0.02, P = 0.75	*r* = 0.06, P = 0.46
Physical therapy for hip OA is not effective	*r* = 0.02, P = 0.81	*r* = 0.04, P = 0.61	*r* = -0.10, P = 0.21	*r* = -0.12, P = 0.11	***r* = -0.29, P<0.01**	*r* = -0.06, P = 0.47	*r* = 0.08, P = 0.30	*r* = 0.06, P = 0.40
Lack of knowledge about guideline	*r* = -0.07, P = 0.34	***r* = -0.21, P<0.01**	***r* = -0.17, P = 0.03**	*r* = -0.07, P = 0.35	*r* = -0.02, P = 0.82	*r* = -0.05, P = 0.51	*r* = -0.03, P = 0.67	*r* = 0.11, P = 0.14
**Patient**								
Patient cannot afford absenteeism at work	*r* = 0.14, P = 0.06	***r* = 0.15, P = 0.05**	*r* = 0.10, P = 0.22	*r* = -0.01, P = 0.99	*r* = 0.06, P = 0.94	*r* = 0.08, P = 0.31	*r* = -0.02, P = 0.81	*r* = 0.03, P = 0.68
Pressure by patient for surgery	*r* = 0.12, P = 0.11	*r* = -0.02, P = 0.84	*r*<-0.01, P = 0.97	*r* = 0.14, P = 0.08	*r* = -0.07, P = 0.36	*r* = 0.03, P = 0.66	*r* = 0.07, P = 0.37	***r* = 0.15, P = 0.05**
**Social context**								
Trained to be reluctant with Glucocorticoid injections^a^	*r* = 0.11, P = 0.16	*r*<0.01, P = 0.10	*r* = 0.13, P = 0.09	*r* = 0.13, P = 0.08	*r* = 0.03, P = 0.68	*r* = 0.12, P = 0.12	*r* = 0.05, P = 0.53	***r* = -0.30, P<0.01**
**Organizational context**								
Clarity on what the patient has done at the physical therapist	*r* = 0.02, P = 0.80	*r* = 0.10, P = 0.20	***r* = 0.17, P = 0.03**	*r* = 0.14, P = 0.06	***r* = 0.30, P<0.01**	***r* = 0.25, P = 0.01**	*r* = 0.04, P = 0.60	*r* = -0.02, P = 0.81
Agreements/ deliberations with primary care (GP, physical therapist, dietician)	*r* = 0.08, P = 0.33	*r* = -0.04, P = 0.65	*r* = 0.03, P = 0.68	***r* = 0.18, P = 0.02**	***r* = 0.20, P<0.01**	*r* = 0.11, P = 0.16	*r* = 0.12, P = 0.13	***r* = 0.17, P = 0.02**
Presence of an obesity clinic	*r* = 0.06, P = 0.42	*r* = 0.06, P = 0.42	*r* = 0.14, P = 0.06	***r* = 0.36, P<0.01**	***r* = 0.20, P = 0.01**	***r* = 0.17, P = 0.02**	***r* = 0.16, P = 0.03**	*r* = 0.02, P = 0.77
Easy communication with a dietician	*r* = -0.06, P = 0.47	*r* = -0.03, P = 0.72	*r* = 0.05, P = 0.55	***r* = 0.29, P<0.01**	*r* = 0.09, P = 0.27	*r* = 0.13, P = 0.09	*r* = 0.09, P = 0.24	*r* = 0.02, P = 0.79
Non-surgical treatments belong to primary care	*r* = -0.01, P = 0.93	*r* = -0.04, P = 0.58	*r* = 0.01, P = 0.94	*r* = -0.06, P = 0.42	***r* = -0.23, P<0.01**	*r* = 0.02, P = 0.84	*r* = 0.10, P = 0.21	*r* = -0.05, P = 0.53
Lack of referral structure to physical therapist	*r* = -0.07, P = 0.37	*r* = -0.02, P = 0.83	*r* = -0.09, P = 0.24	*r* = 0.01, P = 0.87	***r* = -0.20, P = 0.01**	*r* = 0.01, P = 0.92	***r* = 0.17, P = 0.03**	*r* = -0.01, P = 0.91
**Economic and political context**								
Availability of non-surgical treatments	*r* = 0.06, P = 0.45	*r* = 0.15, P = 0.06	*r* = 0.14, P = 0.06	***r* = 0.20, P = 0.01**	***r* = 0.16, P = 0.04**	*r* = 0.14, P = 0.06	*r*<-0.01, P = 0.97	*r* = 0.02, P = 0.80
A consult at a dietician is not covered by insurance	*r* = 0.01, P = 0.87	*r* = 0.05, P = 0.54	*r* = 0.07, P = 0.36	*r* = 0.02, P = 0.83	*r* = -0.01, P = 0.87	*r* = 0.13, P = 0.10	***r* = 0.19, P = 0.01**	*r* = 0.10, P = 0.19

*r* = Spearman rank correlation

In bold: P-values ≤ 0.05

^a^ Only for patients with knee OA

Only a few of these barriers and facilitators were independently and significantly associated with prescription of non-surgical treatments in the multivariable logistic regression analysis (Table 6). Lack of knowledge about the guideline was associated with a decreased prescription of lifestyle advice. Agreements/ deliberations with primary care (GP, physical therapist, dietician) and easy communication with a dietician were both associated with increased prescription of dietary therapy. For acetaminophen, NSAIDs, and glucocorticoid injections, the belief in the efficacy of these treatments was associated with increased prescription. On the other hand, the belief that physical therapy for hip OA is not effective and that there is no effect when there is an obvious loss of cartilage was associated with decreased prescription of physical therapy.

**Table 6:** The independent effect of barriers and facilitators reported by orthopaedic surgeons for prescription of non-surgical treatments (multivariable analyses).

Non-surgical treatment	Provided, yes (%)	Barrier (B) or facilitator (F)	Odds ratio (95% Confidence interval)	p-value
Education about OA	87	-	-	-
Education about different treatment options	95	-	-	-
Lifestyle advice	98	Lack of knowledge about guideline	0.03 (0.001–0.50)	0.015
(Referral to) dietary therapy	28	Easy communication with a dietician	6.21 (1.48–26.10)	0.013
		Agreements/ deliberations with primary care (GP, physical therapist, dietician)	2.41 (1.05–5.53)	0.037
Referral to) physical therapy	54	Presence of an obesity clinic	4.12 (1.42–11.96)	0.009
		Clarity on what the patient has done at the physical therapist	2.42 (1.07–5.47)	0.034
		Physical therapy for hip OA is not effective	0.43 (0.20–0.92)	0.029
		No effect of physical therapy when there is an obvious loss of cartilage	0.39 (0.18–0.82)	0.013
Acetaminophen	64	Acetaminophen has only a few side effects	7.99 (2.16–29.64)	0.002
		Important to try non-surgical treatments first	5.15 (1.16–22.87)	0.031
		Patients benefit from Acetaminophen	5.14 (1.80–14.72)	0.002
		No effect of physical therapy when there is an obvious loss of cartilage	0.23 (0.09–0.58)	0.002
NSAIDs	59	Patients benefit from NSAIDs	5.96 (2.45–14.52)	<0.001
		Pressure by patient for surgery	3.92 (1.63–9.45)	0.002

Only barriers and facilitators with P-values ≤ 0.05 are shown in the table

## Discussion

This study revealed barriers and facilitators for non-surgical treatment use in patients with hip and knee OA in orthopaedic practice. Most of the identified facilitators and barriers reported by orthopaedic surgeons reflect views on the effectiveness of non-surgical treatments. For example, the barriers “Physical therapy for hip OA is not effective” or “No effect of physical therapy when there is an obvious loss of cartilage” were associated with decreased prescription of physical therapy. The facilitators “Patients benefit from Acetaminophen, NSAIDs or Glucocorticoid injections” were associated with an increased prescription of Acetaminophen, NSAIDs and Glucocorticoid injections, respectively. This means that an intervention to improve non-surgical treatment use may be targeted at trying to change the beliefs regarding the efficacy of non-surgical treatments among orthopaedic surgeons.

In addition, most of the barriers and facilitators reported by patients that were associated with the use of non-surgical treatment use reflect the importance of their environment e.g. “People in my environment had positive experiences with surgery” and “Advice of people in my environment to keep on moving”. Another study found that “help by others” was a facilitator for the use of analgesics in patients with knee OA [10]. Thus it seems to be important to involve patients’ environment (e.g. partners or other family members) so that they all understand the importance of non-surgical treatments, such as exercises and losing weight, and support the patient in using these treatments.

Previous studies focused on patients’ characteristics or on a specific treatment, whereas the present study adds that the patients’ environment and the views of orthopaedic surgeons on the effectiveness of non-surgical treatments play an important role in the use of these treatments. This is consistent with the barrier reported by patients reflecting the view of their health care provider: “Lack of trust in non-surgical treatments”, “Preference of practitioner for surgery” and “Too much loss of cartilage to use non-surgical treatments”. Furthermore, in our previous study only 54% of the orthopaedic surgeons reported that they referred patients to a physical therapist if a patient did not have that before [13]. This could partly be explained by the barriers reported by orthopaedic surgeons that were significantly associated with a decreased prescription of physical therapy: “Physical therapy for hip OA is not effective” and “No effect of physical therapy when there is an obvious loss of cartilage”. This shows that orthopaedic surgeons do not always believe in the effectiveness of physical therapy, even though evidence based guidelines do advice this [28]. Orthopaedic surgeons also perceived many barriers and facilitators regarding communication with primary care. In addition, a good collaboration between health care providers was associated with reported increased use of dietary therapy, as reported by patients. Therefore, it seems that clear referral criteria are needed between primary and hospital care, and agreements about the organization of care, for example how the physical therapist treats a patient. Focusing on dietary therapy, it appeared that “Agreements/ deliberations with primary care (GP, physical therapist, dietician)” and “Easy communication with a dietician” may facilitate the prescription of this treatment. Therefore, strategies to improve the prescription of these non-surgical treatments should also focus on the communication between orthopaedic surgeons and other health care providers, clear referral criteria and agreement about the organization of care, apart from changing the beliefs of orthopedic surgeons regarding the effectiveness of these non-surgical treatments.

This study has some limitations. First, because of the retrospective nature of our study and the reliance on self-reported data, it is susceptible to recall bias. To reduce this influence we only included patients who had a TKA or THA no longer than 12 months ago, or scheduled for surgery within the next 3 months. Second, the use of an internet-based survey could have induced selection bias. It is possible that more elderly persons do not have internet or an email address compared to younger persons. Indeed, the average age of patients with OA is 68 years [29] whereas the average age of our population was slightly lower, i.e. 64 (SD 7.7) years. Furthermore, response bias may have occurred because orthopaedic surgeons with an interest in non-surgical treatments may be more motivated and willing to participate and may perceive other barriers or facilitators. However, our response rate is comparable or higher than found in other online surveys among orthopaedic surgeons regarding different subjects [30–32]. Given the equal spread of respondents across the Netherlands, we think we will have captured all regions and thereby a rather complete view of both barriers and facilitators. Another limitation is that patients could answer “not applicable to my situation” in our survey. Although we explained to patients to choose this option only when they did not visit for example an exercise therapist when referring to barriers and facilitators for visiting an exercise therapist, it seems that this has been misunderstood. Despite this explanation and a previous pilot test of the questionnaire, we feel that patients misinterpreted this category. Therefore, we assumed that a patient would have selected an item if the patient had felt strongly about that item and interpreted “not applicable” as “not important”. Treating the answers “not applicable” as missing gave similar results in the univariate analyses (data not shown), which confirms the robustness of our results.

Strength of this study is that barriers and facilitators in the survey were identified during interviews with patients and orthopaedic surgeons in regions with low and high surgery rates. This ensures that the survey does not test the authors’ personal hypothesis but represents a rather complete set of possible barriers and facilitators based on existing frameworks. Another strong point is the finding that barriers and facilitators are independently associated with the use of non-surgical treatments. This ensures that identified barriers and facilitators are relevant to optimize of the use of non-surgical treatments. Still, the results of these multivariable regression analyses should be interpreted carefully, since answering categories were dichotomized [33]. For proper interpretation of results, the percentage using each non-surgical treatment, association of each barrier and facilitator and the multivariable analyses should all be taken into account.

Insight into barriers and facilitators is essential to optimize the use and prescription of non-surgical treatments. Previous studies that tested implementation strategies all conclude that a prior inventory of barriers and facilitators to develop a tailored implementation strategy is useful and can confirm whether barriers differ between settings [34–36]. Such a prior inventory thereby reduces the number of costly trials evaluating different implementation strategies [34,37,38]. Although previous studies already explored barriers and facilitators for the use of non-surgical treatments, these studies were performed in other settings, did not include all barriers/ facilitators and their influence on different non-surgical treatments, and were mostly focused on the patient level thereby ignoring the influence of professionals and organizations. A different setting may result in another strategy given the results from the present study e.g. if the beliefs regarding the effectiveness of non-surgical treatments differ between primary care and orthopaedic practice. The next step will be the development of an implementation strategy based on all identified barriers and facilitators both on the patient, professional and organizational level, which will be presented to the Dutch Orthopaedic Association to be implemented in clinical practice. Future studies should show whether this strategy is effective in improving the use and prescription of non-surgical care as well as patient outcomes.
